# Comparative systematic review and meta-analysis of pregnancy outcomes after kidney transplantation

**DOI:** 10.3389/frtra.2025.1689018

**Published:** 2025-10-13

**Authors:** Stergios Bobotis, Giorgos Mavrommaths, Vassilios Papalois

**Affiliations:** ^1^ Department of Obstetrics and Gynaecology, Maidstone and Tunbridge Wells NHS Foundation Trust, Royal Tunbridge Wells, United Kingdom; ^2^ Department of Gynecology, General Anti-cancer Oncological Hospital “Agios Savvas”, Athens, Greece; ^3^ Department of Surgery and Cancer, Imperial College London, London, United Kingdom

**Keywords:** kidney transplantation, pregnancy outcomes, pre-eclampsia, gestational hypertension, systematic review, meta-analysis

## Abstract

**Introduction:**

Advancements in transplant medicine have increased the incidence of pregnancy among kidney transplant recipients. These pregnancies, however, carry elevated maternal and neonatal risks, warranting comprehensive outcome evaluation.

**Materials and methods:**

To compare key maternal and neonatal outcomes in pregnancies following kidney transplantation with those in healthy pregnancies. A systematic search of MEDLINE, Embase, and PubMed was conducted up until December 2024. Comparative prospective and retrospective observational studies reporting maternal or neonatal outcomes in pregnancies among kidney transplant recipients and healthy controls. Risk of Bias in Non-Randomized Studies of Interventions (ROBINS-I) was used for quality assessment. Random-effects meta-analyses were conducted to calculate pooled odds ratios (ORs) with 95% confidence intervals (CIs) and heterogeneity (*I*^2^). Sensitivity analysis explored the impact of study design and bias.

**Results:**

Eight studies encompassing 893 pregnancies post-kidney transplantation were included. Relative to healthy pregnancies, kidney-transplant recipients showed markedly higher odds of pre-eclampsia (OR: 10.17, 95% CI: 4.25–24.35; *I*^2^ = 86%), gestational hypertension (OR: 7.40, 95% CI: 2.20–24.86; *I*^2^ = 84%) and preterm birth (OR: 13.65, 95% CI: 4.79–38.92; *I*^2^ = 96%). Caesarean delivery (OR: 3.95, 95% CI: 1.67–9.31; *I*^2^ = 93%) and fetal mortality (OR: 4.84, 95% CI: 1.33–17.57; *I*^2^ = 79%) were also higher, whereas gestational diabetes did not differ (OR: 1.06, 95% CI: 0.67–1.67; *I*^2^ = 0%). Sensitivity analyses confirmed the elevated risks of pre-eclampsia and preterm birth, whereas the associations with caesarean section and fetal mortality did not remain statistically significant after adjustment for study quality.

**Conclusions:**

Pregnancies following kidney transplantation are associated with significantly increased maternal and neonatal risks. These findings underscore the need for specialized antenatal care and further large-scale prospective studies to optimize outcomes and inform clinical guidelines.

## Key message

Pregnancy after kidney transplantation remains high-risk, with significantly increased odds of pre-eclampsia, hypertension, and preterm birth. This meta-analysis reinforces the need for tailored antenatal care and improved risk stratification to safeguard maternal and neonatal outcomes.

## Introduction

Pregnancy in kidney transplant recipients presents a complex interplay between maternal health, graft viability, and fetal outcomes. Advances in transplant medicine have significantly improved survival and quality of life, leading to an increasing number of women of childbearing age considering pregnancy post-transplant. However, pregnancy in this population poses unique challenges due to the physiological stress imposed on the transplanted kidney, including increased glomerular filtration rate (GFR) and vasodilation (1, 2).

End-stage renal disease (ESRD) is strongly associated with impaired fertility, with over 90% of women on dialysis experiencing amenorrhea or irregular menstrual cycles (3). This is primarily due to hypothalamic-pituitary-ovarian axis dysfunction (3, 4), which often resolves following kidney transplantation (5). Ovulation can resume within months post-transplant, with menstrual cycles normalizing in most women within a year (3, 5). Given the risks associated with pregnancy in transplant recipients, careful timing of conception is essential. The primary objective of delaying pregnancy post-transplant is to ensure stable graft function while minimizing immunosuppression to reduce the risk of infectious complications. Women are generally advised to conceive only if they meet criteria such as a serum creatinine <133 μmol/L, absence of significant proteinuria, no recent rejection episodes, and well-controlled comorbidities (e.g., hypertension, diabetes) (6). While pregnancy as early as six months post-transplant may be considered for women meeting these criteria, pregnancies in transplant recipients remain high-risk, necessitating multidisciplinary management by transplant and obstetric specialists (2, 7).

While pregnancy is feasible and often successful in women with a well-functioning graft, it remains associated with higher complication rates compared to the general population. These include an increased risk of pre-eclampsia, preterm birth, and graft dysfunction (2, 7, 8). Fetal complications, such as small-for-gestational-age (SGA) infants and low birth weight, are also more prevalent in this group (7, 8). Additionally, immunosuppressive therapy, essential for graft maintenance, carries potential maternal and fetal risks, including teratogenic effects and increased susceptibility to infections (9).

Given the rising number of pregnancies post-transplant, a comprehensive understanding of the risks and outcomes associated with such pregnancies is essential. This meta-analysis synthesizes existing evidence on maternal and fetal outcomes in kidney transplant recipients.

## Methods

This review was reported based on the “Preferred Reporting Items for Systematic Reviews and Meta-Analyses” (PRISMA) guidelines. The protocol was registered in PROSPERO (CRD420250655797) (10).

### Literature search

A literature search was carried out independently by two reviewers (SB, GM) on MEDLINE, Embase, and PubMed from their inception until December 2024. A search strategy containing the following key terms was performed: (((pregnancy[MeSH Terms]) OR (pregnan*)) OR (obstetric)) AND (kidney transplantation[MeSH Terms]) AND (((outcomes) OR (assessment, patient outcome[MeSH Terms])) OR (complication)). A manual search of citations of the included studies and published systematic reviews was also conducted.

### Eligibility criteria

Eligible studies consisted of observational studies comparing pregnancies of kidney transplant recipients (post-transplant pregnancy; PTP) to pregnancies of healthy women with no history of transplantation (Pregnancy only; *P* only). The study design comprised of solely comparative observational studies. Any non-English articles identified were translated and extracted, if appropriate. Single-arm observational studies, systematic reviews, non-peer reviewed articles, and conferences abstract and presentations were excluded.

### Quality assessment

The included studies were assessed for their quality of data using the Risk Of Bias In Non-randomized Studies of Interventions (ROBINS-I) tool. This was carried out by two authors independently (SB, GM) and discrepancies were resolved by a third independent author (VP).

### Data extraction and handling

Data extraction was performed using MS Excel 2018 by two authors (SB, GM) independently. The following were extracted: number of patients, mean maternal age, BMI, parity, time since transplant, pregnancy outcomes, and immunosuppression used.

### Definition of outcomes

The outcomes explored in this study involved obstetric outcomes for the populations of post-transplant pregnancies and normal pregnancies. These were: pre-term birth (PTB) (delivery before 37 completed weeks of gestation), pre-pregnancy hypertension (HTN) (chronic hypertension, non-pregnancy related), gestational hypertension (hypertension identified after 20 weeks of gestation), pre-eclampsia, caesarean section (CS), fetal mortality (stillbirth or early perinatal death <24 h), and gestational diabetes mellitus (GDM).

### Statistical analysis

The main statistical analysis included comparison of post-transplant pregnancies (PTP) and pregnancy only (P) study groups across all outcomes.

Data was analyzed using Cochrane RevMan (Review Manager) software (RevMan, Copenhagen: The Nordic Cochrane Centre, the Cochrane Collaboration, 2008). Odds ratios (OR) were calculated using the Generic Inverse Variance method using a random effect model. For each included study, odds ratios were calculated when only raw event data were available. For such studies and studies that directly reported ORs, either adjusted or unadjusted, the log-transformed ORs and their standard errors (SEs) were calculated from the reported confidence intervals (CI). When both adjusted and unadjusted ORs were reported, only adjusted ratios were extracted. Statistical heterogeneity was investigated using *χ*^2^ test (*P* < 0.10 was significant heterogeneity), and *I*^2^ and *τ*^2^ were used for quantifying the heterogeneity. Specifically, moderate heterogeneity was defined as *I*^2^ values ranging from 30% to 49%, and high heterogeneity as *I*^2^ values ranging from 50% or more.

Additionally, a sensitivity analysis was conducted to assess the potential confounding effects of patient characteristics. Specifically, the meta-analysis was repeated after excluding studies that either lacked matched control groups or failed to adjust for confounding variables in their analyses.

## Results

### Study characteristics

We identified eight studies, published between 2002 and 2024, eligible for the systematic review. Regarding their geographical distribution, seven were conducted in Europe and one in the United States of America.

### Patient characteristics

Out of the total pregnant patients available in these studies, we identified 893 patients who met our criteria for post-transplant pregnancy patients. Patients included in these studies had a mean age of 31 years of age. By further differentiation into categories, post-transplant pregnant women had a mean age of 31.2 (±2.3), while pregnant only patients had a mean age of 30.4 (±3.18).

Regarding gestational delivery age (GA), post-transplant women reached a mean gestational age of 35.83 (± 0.68). On the other side, pregnant only women had a mean gestational age at delivery of 39.45 (±0.47). Concerning the birth weight, in the post-transplant group, the weight at birth was a mean of 2,518.17 (±156.67), while in pregnant only group, the mean weight was 3,418.3 (±127.69).

Data regarding immunosuppression regimes received by the post-transplant patients showcased that the most common was prednisolone (82%, 313/381) followed by cyclosporine (24%, 98/406), tacrolimus (38%, 136/362), azathioprine (61%, 133/219) and Mycophenolate Mofetil (3%, 5/148) (Supplementary Table S1).

### Pregnancy outcomes

**Figure 1:**
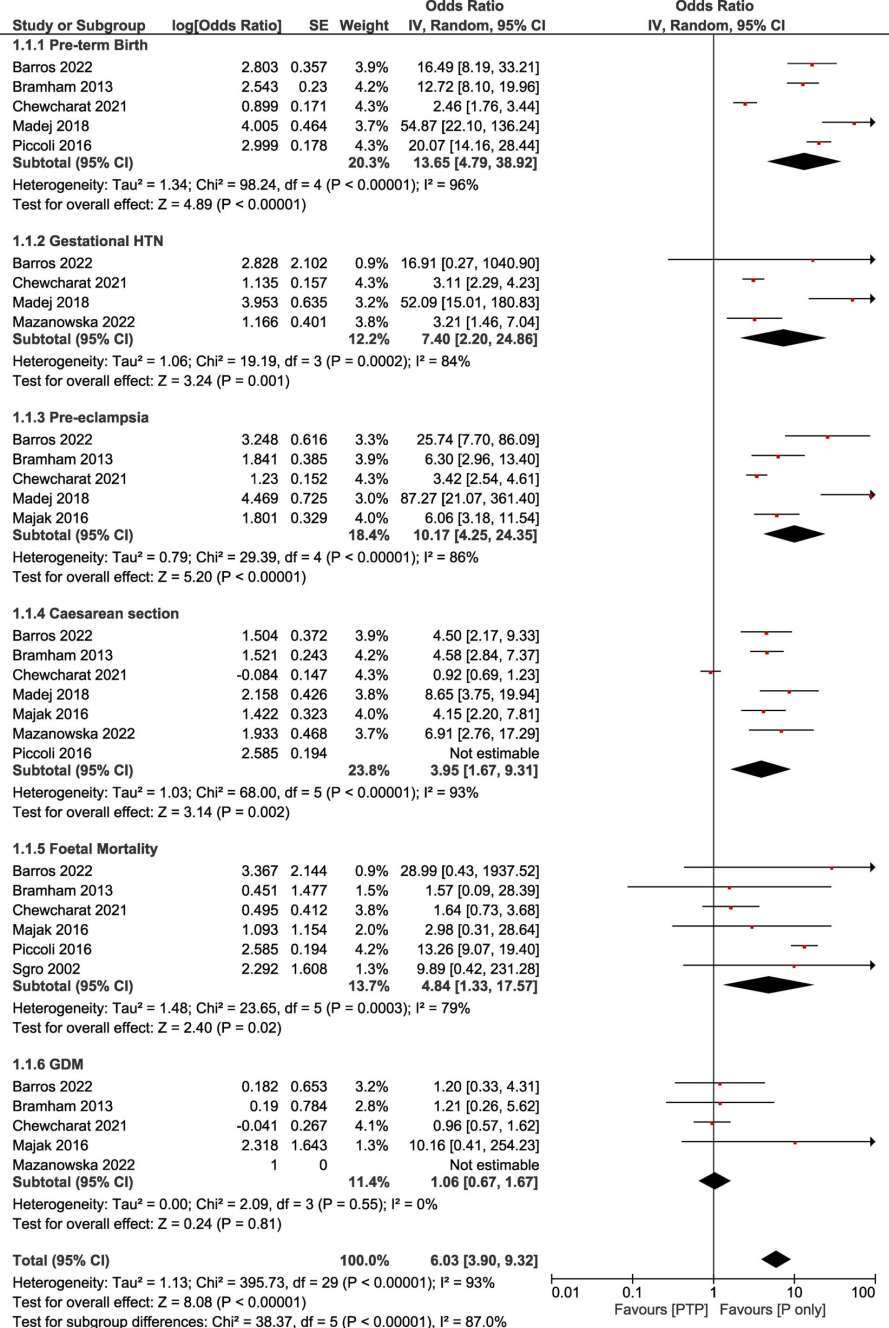
Forest plot demonstrating the results of the main analysis (PTP vs. *P* only) on pregnancy outcomes.

Our main analysis comparing the post-transplant pregnancy women group vs. the pregnant only women group showed that pre-term birth was more prominent in the post-transplant pregnancy group compared to the pregnancy only one (OR: 13.65, 95% CI: 4.79–38.92; *I*^2^ = 96%, *τ*^2^ = 1.34). Similarly, the rate of gestational hypertension was more prominent in the PTP group in comparison with the Pregnancy only group (OR: 7.40, 95% CI: 2.20–24.86; *I*^2^ = 84%, *τ*^2^ = 0.46). Additionally, the pre-eclampsia rate was increased among pregnant patients after transplantation compared to the pregnancy only group (OR: 10.17, 95% CI: 4.25–24.35; *I*^2^ = 86%, *τ*^2^ = 0.79). Moreover, great discrepancy was observed between the number of caesarean sections between the two studied groups (OR: 3.95, 95% CI: 1.67–9.31; *I*^2^ = 93%, *τ*^2^ = 1.03). Following the same trend, fetal mortality was more prominent in the PTP group compared to the pregnancy only one (OR: 4.84, 95% CI: 1.33–17.57; *I*^2^ = 79%, *τ*^2^ = 1.48). Lastly, the rate of gestational diabetes mellitus was not statistically different between the patients of the two studied groups (OR: 1.06, 95% CI: 0.67–1.67; *I*^2^ = 0%, *τ*^2^ = 0) (Table 1, Figure 1).

**Table 1:** Summary of findings for the sensitivity analysis (PTP vs. pregnancy only).

Outcome	No of studies	Odds Ratio (95% CI) random effects model (inverse variance method)	*I* ^2^	*τ* ^2^
Pre-term Birth	5	13.65 [4.79, 38.92]	96%	1.34
Gestational HTN	4	7.40 [2.20, 24.86]	84%	1.06
Pre-eclampsia	5	10.17 [4.25, 24.35]	86%	0.79
Caesarean Section	7	3.95 [1.67, 9.31]	93%	1.03
Foetal Mortality	6	4.84 [1.33, 17.57]	79%	1.48
GDM	5	1.06 [0.67, 1.67]	0	0.00

95% CI, 95% confidence interval; PTP, post-transplant pregnancy; P, pregnancy only; HTN, hypertension; GDM, gestational diabetes mellitus.

### Sensitivity analysis

**Figure 2:**
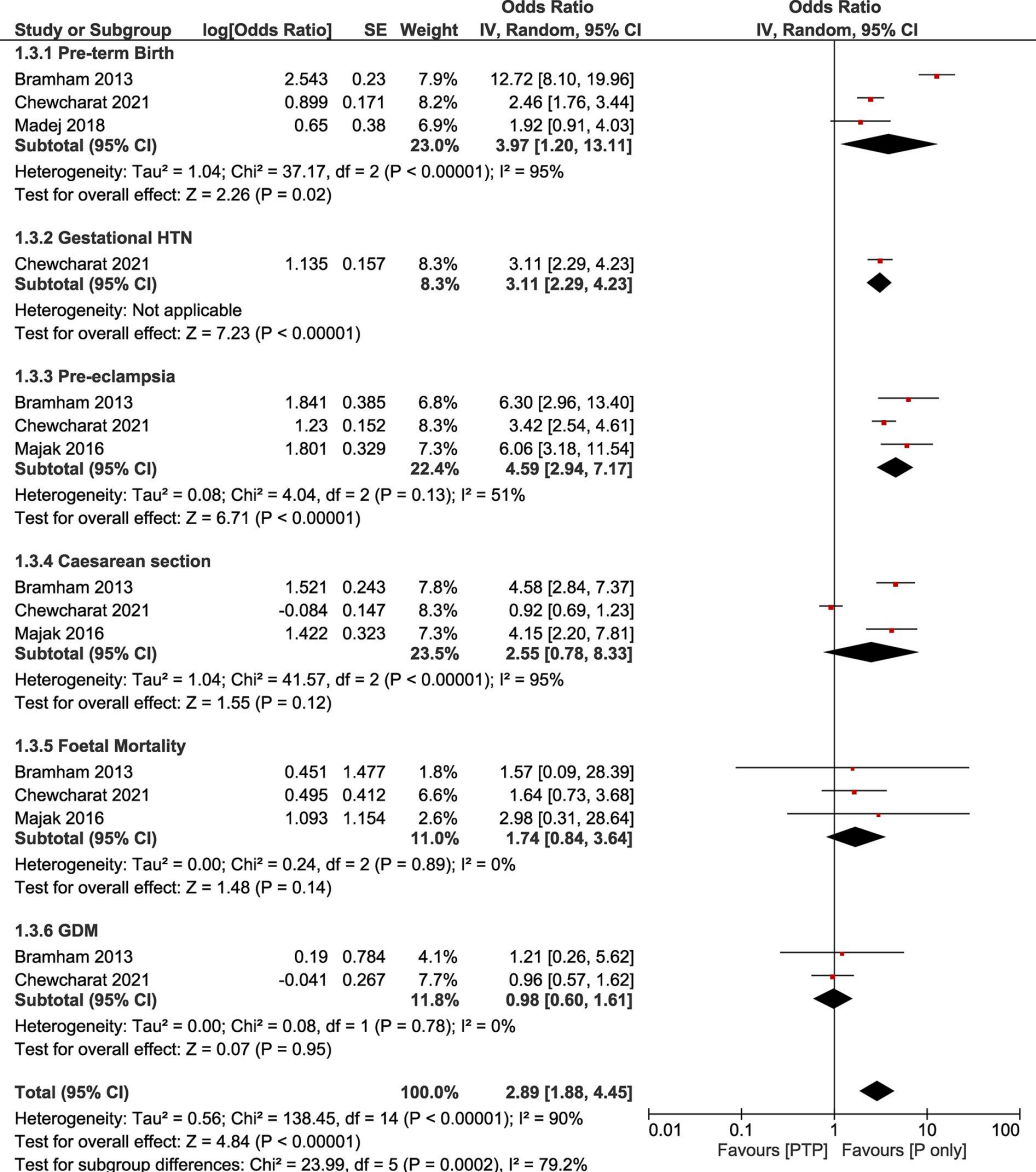
Forest plot demonstrating the results of the sensitivity analysis (PTP vs. *P* only) on pregnancy outcomes.

The sensitivity analysis revealed that statistical significance between groups was present for the outcomes for pre-term birth (OR: 3.97, 95% CI: 1.20–13.11; *I*^2^ = 95%, *τ*^2^ = 1.04) and pre-eclampsia (OR: 4.59, 95% CI: 2.94–7.17; *I*^2^ = 51%, *τ*^2^ = 0.08). In contrast to the main analysis, no statistically significant difference was found for caesarean sections (OR: 2.55, 95% CI: 0.78–8.33; *I*^2^ = 95%, *τ*^2^ = 1.04), GDM (OR: 0.98, 95% CI: 0.60–1.61; *I*^2^ = 0%, *τ*^2^ = 0), and fetal mortality (OR: 1.74, 95% CI: 0.84–3.64; *I*^2^ = 0%, *τ*^2^ = 0). Our sensitivity analysis showcased high levels of heterogeneity for the studied outcomes with pre-eclampsia rates for this group showcasing lower heterogeneity (Table 2, Figure 2).

**Table 2:** Summary of findings for the comparative analysis (PTP vs. pregnancy only).

Outcome	No of studies	Odds Ratio (95% CI) random effects model (inverse variance method)	*I* ^2^	*τ* ^2^
Pre-term Birth	3	3.97 [1.20, 13.11]	95%	1.04
Gestational HTN	1	3.11 [2.29, 4.23]	Not applicable	Not applicable
Pre-eclampsia	3	4.59 [2.94, 7.17]	51%	0.08
Caesarean Section	3	2.55 [0.78, 8.33]	95%	1.04
Foetal Mortality	3	1.74 [0.84, 3.64]	0	0.00
GDM	2	0.98 [0.60, 1.61]	0	0.00

95% CI, 95% confidence interval; PTP, post-transplant pregnancy; P, pregnancy only; HTN, hypertension; GDM, gestational diabetes mellitus.

### Risk of bias

Risk of bias assessment of the included studies revealed one study with low risk, five with moderate risk, and two with serious risk of bias. Overall, all studies were judged to have at least moderate risk of bias (Supplementary Table S2).

## Discussion

### Main findings

This comparative systematic review and meta-analysis confirm that pregnancy is feasible after kidney transplantation but is associated with increased maternal and fetal risks. Notably, pre-eclampsia was strongly linked to post-transplant pregnancies (OR: 4.59, 95% CI: 2.94–7.17), with significantly higher rates of gestational hypertension and preterm birth in the exposed group. These findings align with existing epidemiological data, including the UK Transplant Pregnancy Registry (UKTPR) (11) which reported a 36% prevalence of gestational hypertension and pre-eclampsia in transplant pregnancies—representing a six-fold increase compared to the general population. Furthermore, two large systematic reviews and meta-analyses have documented similar rates of pre-eclampsia, with pooled prevalence estimates of 21.5% (95% CI, 18.5–24.9) (7) and 27.0% (95% CI, 25.2–28.9) (8), reinforcing the heightened risk in this population. In contrast, other studied outcomes, including caesarean section, fetal mortality, and gestational diabetes mellitus (GDM), did not differ significantly between post-kidney transplant pregnancies and healthy pregnancies. It is notable that in our study, the associations with caesarean section and fetal mortality observed in the main analysis did not remain statistically significant in sensitivity analyses. This suggests that these findings may be more vulnerable to confounding and study heterogeneity and should therefore be interpreted with caution. In contrast, the associations with pre-eclampsia and preterm birth remained consistent, reinforcing the robustness of these outcomes.

This study highlights the increased obstetric and fetal risks involved in pregnancies following kidney transplantation. It should be noted that distinguishing between gestational hypertension and pre-eclampsia in renal transplant recipients poses a clinical challenge. Blood pressure commonly rises in late pregnancy which usually exacerbates pre-existing proteinuria secondary to hyperfiltration. Clinical signs of fluid overload are not very helpful as they are usually co-existent in kidney transplant patients and hyperuricemia is not reliable as immunosuppressants, such as calcineurin inhibitors, increase uric acid levels (11, 12) Pre-pregnancy hypertension is also a strong predisposing factor for the development of pre-eclampsia in transplant recipients (13–15). Transplanted kidneys have altered vascular regulation due to surgical factors and prior ischemia-reperfusion injury. This predisposes recipients to increased blood pressure sensitivity before and during pregnancy (14, 16, 17). These pathophysiological mechanisms have been extensively described in prior literature (18, 19).

Hypertensive disorders of pregnancy also significantly increase risks of maternal and fetal complications including pre-term birth, intra-uterine growth restriction, and fetal mortality. Shah et al., a large systematic review that analyzed 87 observational studies on outcomes in pregnancy after a kidney transplant found a pre-term birth incidence of 43% and a mean gestational age of 34.9 weeks, findings similar to this comparative study (7). Premature deliveries are highly associated with hypertensive disorders in pregnancy, which are evidently increased in renal transplant recipients, reported as high as 40%–60% against the 5%–10% of the general population (20). Use of immunosuppressants increases risks of infections as well, particularly UTIs, occurring in up to 42% of such pregnancies (21) and therefore increasing chances of pre-term birth. Although fetal mortality was found to be higher when compared to normal pregnancies, national data does not suggest significant differences in live births (20). Nonetheless, neonatal intensive care unit (NICU) admissions are significantly increased, primarily due to complications such as respiratory distress syndrome and infection risk, with reports suggesting rates as high as 20% in this population (22). These findings highlight the need for close neonatal monitoring and individualized postnatal care in transplant pregnancies.

Gestational diabetes mellitus (GDM) would be expected to be prevalent among kidney transplant recipients due to the diabetogenic effects of immunosuppressive medications, particularly tacrolimus and corticosteroids (23, 24). However, our sensitivity analysis did not reveal a significant difference in GDM prevalence between transplant recipients and the general obstetric population. Interestingly, Shah et al. reported substantial geographical variation in GDM prevalence, with the highest rates observed in Europe (8.9%), a finding recently corroborated by Mustafa et al. (7, 25). This variation may be attributable to differences in diagnostic criteria, ethnic predispositions, and the heterogeneity of immunosuppressive regimens across regions (26, 27). Overall, GDM prevalence in kidney transplant recipients ranges from 3% to 12%, aligning closely with rates observed in the general population (8).

Although the anatomical position of a renal allograft should not prompt clinicians to perform caesarean sections over normal vaginal deliveries (28), it is evident that in clinical practice the opposite is true (11, 20). It is true that there is global variation in the rates of CS being performed with lower rates reported in Europe (50%–60%) compared to North America (70%–80%) (29). High rates of maternal and fetal complications, such as hypertensive disorders and pre-term birth that are highly prevalent in the studied population could explain the volume of CS performed, but such an association warrants further study.

### Strengths and limitations

To our knowledge, this is the first comparative systematic review and meta-analysis investigating obstetric and fetal outcomes in women with kidney transplants. By utilizing matched controls, we aimed to minimize the impact of individual patient characteristics and confounding variables. A sensitivity analysis, excluding studies that did not match controls or adjust for confounding factors, further strengthened the robustness of our findings. However, pre-pregnancy hypertension is well-documented as a major risk factor for obstetric complications, yet only one study accounted for this variable in its analysis (30). From the observational studies, risk-of-bias score based on ROBINS-I tool was moderate overall, indicating mostly poor-quality studies. The presence of confounding factors and the selection of reported results were the most commonly affected domains in the risk of bias assessment. As a result, our findings may be subject to bias, particularly given the limited number of studies remaining after sensitivity analysis, potentially reducing statistical power. A further limitation concerns the reporting of hypertensive disorders. While some studies distinguished between chronic (pre-pregnancy) hypertension and gestational hypertension, others did not, which may have influenced the pooled estimates. In addition, granular data on comorbidities and management strategies were often lacking. Most studies did not specify whether women were on antihypertensive therapy post-transplant or provide sufficient detail to assess outcomes by blood pressure control. Similarly, the primary cause of kidney disease was inconsistently reported, limiting exploration of its potential impact on maternal and neonatal outcomes. Nonetheless, our results align with national registry data and previous systematic reviews, reinforcing their validity. While heterogeneity was observed across all included studies (30–37), pre-eclampsia remained a consistent finding, with moderate heterogeneity (*I*^2^ = 52%).

## Conclusion

Pregnancy following kidney transplantation remains feasible but high-risk, with significantly increased odds of hypertensive disorders and preterm birth. These findings underscore the importance of early risk stratification, tailored antenatal care, and coordinated multidisciplinary management. Future prospective studies are essential to better understand the long-term outcomes for both mothers and infants, and to guide evidence-based clinical practice in this growing patient population.
