# Supplementary material for: Comparative systematic review and meta-analysis of pregnancy outcomes after kidney transplantation
Source: Front Transplant. 2025 Oct 13;4:1689018. doi: 10.3389/frtra.2025.1689018 (PMC12554720; doi:10.3389/frtra.2025.1689018)
Supplement: Supplementary file 1 [file Table1.docx]

**Table S1.** Summary of the included studies.

|  | **Author**  **Country**  **Year** | **No of patients (total)** | **Age (mean/median)** | | **No of patients** | | **Time since transplant (months)** | **Obstetric Outcomes** | | | | | | | | | | | | | | |
| --- | --- | --- | --- | --- | --- | --- | --- | --- | --- | --- | --- | --- | --- | --- | --- | --- | --- | --- | --- | --- | --- | --- |
|  |  |  |  |  |  |  |  | **PTB** | | **Pre-pregnancy HTN** | | **Gestational HTN** | | **Preeclampsia** | | **CS** | | **Foetal Mortality** | | **Foetal Macrosomia/GDM** | | |
|  |  |  | **PTP** | **P** | **PTP** | **P** | **PTP** | **PTP** | **P** | **PTP** | **P** | **PTP** | **P** | **PTP** | **P** | **PTP** | **P** | **PTP** | **P** | **PTP** | **P** |  |
| **PTP vs**  **P only** | A.Madej  Poland  2018 | 217 | 29.6 (mean) | 26.6 (mean) | 47 | 170 | n/a | 33/47 | 7/175 | 37/47 | 7/170 | 18/47 | 2/170 | 16/47 | 1/170 | 41/47 | 75/170 | n/a | n/a | 0/47 | 1/175 |  |
|  | B.Majak  Norway  2016 | 357 | 30.3 (mean) | 26 (mean) | 119 | 238 | n/a | 45/119 | 17/238 | 38/119 | 0/238 | 7/119 | 0/238 | 48/119 | 14/238 | 77/119 | 33/238 | 5/119 | 1/238 | n/a | n/a |  |
|  | Barros  Portugal  2022 | 237 | 30.6 (mean) | 32.5 (mean) | 37 | 200 | 54.84 | 24/35 | 21/194 | 25/37 | 2/200 | 1/35 | 0/200 | 11/35 | 4/194 | 24/35 | 60/194 | 2/35 | 0/200 | 4/43 | 18/194 |  |
|  | Sgro  Canada  2002 | 132 | 28 (mean) | n/a (mean) | 44 | 88 | n/a | n/a | n/a | n/a | n/a | n/a | n/a | n/a | n/a | n/a | n/a | 4/44 | 0/44 | n/a | n/a |  |
|  | Mazanwska  Poland  2022 | 137 | 35 (median) | 34.1 (median) | 27 | 110 | 106.8 | n/a | n/a | 21/27 | 0/110 | 6/27 | 9/110 | n/a | n/a | 21/27 | 37/110 | n/a | n/a | 5/27 | 7/110 |  |
|  | Chewcharat  USA  2021 | 5405794 | n/a (median) | n/a (median) | 295 | 5405499 | n/a | 88/295 | 361609/5405499 | n/a | n/a | 115/295 | 312843/5405499 | 102/295 | 238925/5405499 | 164/295 | 1789088/5405499 | 10/295 | 36551/5405499 | 20/295 | 343979/5405499 |  |
|  | Braham  UK  2013 | 1465 | 32 (mean) | 32 (mean) | 105 | 1360 | 60 | 51/108 | 114/1375 | 57/101 | n/a | n/a | n/a | 23/95 | 17/477 | 61/95 | 326/1360 | 1/108 | 10/1375 | 3/95 | 26/1360 |  |
|  | Picolli  Italy  2016 | 1637 | 32.8 (mean) | 31.2 (mean) | 219 | 1418 | 59 | 106/185 | 89/1418 | n/a | n/a | n/a | n/a | n/a | n/a | 155/187 | 379/1418 | 1/189 | 3/1418 | n.a | n/a |  |

| **Author**  **Country**  **Year** | **No of patients (total)** | **No of patients** | | **Time since transplant (months)** | **Immunosuppression (PTP)** | | | | |
| --- | --- | --- | --- | --- | --- | --- | --- | --- | --- |
|  |  | **PTP** | **P** | **PTP** | **Cyclosporine** | **Tacrolimus** | **Azathioprine** | **Mycophenolate Mofetil** | **Prednisolone** |
| A.Madej  Poland  2018 | 217 | 47 | 170 | n/a | n/a | n/a | n/a | n/a | n/a |
| B.Majak  Norway  2016 | 357 | 119 | 238 | n/a | n/a | n/a | n/a | n/a | n/a |
| Barros  Portugal  2022 | 237 | 37 | 200 | 54.84 | 27/43 | 2/43 | 32/43 | 1/43 | 38/43 |
| Sgro  Canada  2002 | 132 | 44 | 88 | n/a | 31/44 | n/a | 39/44 | n/a | 44/44 |
| Mazanwska  Poland  2022 | 137 | 27 | 110 | 106.8 | 7/27 | 19/27 | 1/27 | n/a | n/a |
| Chewcharat  USA  2021 | 5405794 | 295 | 5405499 | n/a | n/a | n/a | n/a | n/a | n/a |
| Braham  UK  2013 | 1465 | 105 | 1360 | 60 | 22/105 | 65/105 | 61/105 | 4/105 | 56/105 |
| Picolli  Italy  2016 | 1637 | 219 | 1418 | 59 | 112/219 | 50/219 | n/a | n/a | 175/219 |

PTP= post-transplant pregnancy, P= pregnancy only, PTB= Preterm Birth

| **Author**  **Year** | **Domain 1: Confounding factors** | **Domain 2: Selection of participants** | **Domain 3: Intervention classification** | **Domain 4: Deviation from intervention** | **Domain 5: Missing data** | **Domain 6: Measurement of outcome** | **Domain 7: Selection of reported result** | **ROBINS-I**  **overall score** |
| --- | --- | --- | --- | --- | --- | --- | --- | --- |
| A.Madej  Poland  2018 | Moderate | Serious | Serious | Moderate | Moderate | Moderate | Serious | Serious |
| B.Majak  Norway  2016 | Moderate | Moderate | Low | Moderate | Low | Moderate | Moderate | Moderate |
| Barros  Portugal  2022 | Moderate | Serious | Moderate | Moderate | Low | Moderate | Moderate | Moderate |
| Sgro  Canada  2002 | Low | Moderate | Low | Low | Low | Moderate | Moderate | Low |
| Mazanowska  Poland  2022 | Low | Moderate | Low | Moderate | Low | Moderate | Moderate | Moderate |
| Chewcharat  USA  2021 | Serious | Serious | Serious | Serious | Moderate | Moderate | Serious | Serious |
| Braham  UK  2013 | Moderate | Low | Low | Low | Moderate | Moderate | Low | Moderate |
| Picolli  Italy  2016 | Moderate | Moderate | Moderate | Low | Low | Moderate | Low | Moderate |

**Table S2.** Risk of bias assessment for observational (ROBINS-I tool) studies

**Figure S1.** PRISMA flowchart.

Inclusion

Eligibility

Screening

Identification

Literature search from 3 electronic databases

(n=1045)

Studies included in the meta-analysis (n=8)

Studies included in the review

(n=8)

Full text screening for eligibility

(n=22)

Full text screening

(n=73)

Articles after the removal of duplicates

(n=758)

Duplicates removed

(n=287)

Articles excluded as irrelevant

(n=685)

Articles removed as irrelevant

(n=51)

Articles removed (n=14)

14 articles without a comparator group

Articles retrieved from references of relevant papers (n=0)
